# Determination of Hg(II) and Methylmercury by Electrothermal Atomic Absorption Spectrometry after Dispersive Solid-Phase Microextraction with a Graphene Oxide Magnetic Material

**DOI:** 10.3390/molecules28010014

**Published:** 2022-12-20

**Authors:** Yesica Vicente-Martínez, María Jose Muñoz-Sandoval, Manuel Hernandez-Cordoba, Ignacio Lopez-Garcia

**Affiliations:** Department of Analytical Chemistry, Faculty of Chemistry, Regional Campus of International Excellence “Campus Mare Nostrum”, University of Murcia, 30100 Murcia, Spain

**Keywords:** mercury, methylmercury, speciation, magnetic dispersive solid-phase microextraction, electrothermal atomic absorption spectrometry

## Abstract

The toxicity of all species of mercury makes it necessary to implement analytical procedures capable of quantifying the different forms this element presents in the environment, even at very low concentrations. In addition, due to the assorted environmental and health consequences caused by each mercury species, it is desirable that the procedures are able to distinguish these forms. In nature, mercury is mainly found as Hg^0^, Hg^2+^ and methylmercury (MeHg), with the latter being rapidly assimilated by living organisms in the aquatic environment and biomagnified through the food chain. In this work, a dispersive solid-phase microextraction of Hg^2+^ and MeHg is proposed using as the adsorbent a magnetic hybrid material formed by graphene oxide and ferrite (Fe_3_O_4_@GO), along with a subsequent determination by electrothermal atomic absorption spectrometry (ETAAS). On the one hand, when dithizone at a pH = 5 is used as an auxiliary agent, both Hg(II) and MeHg are retained on the adsorbent. Next, for the determination of both species, the solid collected by the means of a magnet is suspended in a mixture of 50 µL of HNO_3_ (8% *v*/*v)* and 50 µL of H_2_O_2_ at 30% *v*/*v* by heating for 10 min in an ultrasound thermostatic bath at 80 °C. On the other hand, when the sample is set at a pH = 9, Hg(II) and MeHg are also retained, but if the solid collected is washed with N-acetyl-L-cysteine only, then the Hg(II) remains on the adsorbent, and can be determined as indicated above. The proposed procedure exhibits an enrichment factor of 49 and the determination presents a linear range between 0.1 and 10 µg L^−1^ of mercury. The procedure has been applied to the determination of mercury in water samples from different sources.

## 1. Introduction

Mercury (Hg) is present in nature as a pure elemental substance (liquid mercury) and as inorganic mercury in the form of salts such as HgS (cinnabar), Hg_2_Cl_2_ (calomel), HgCl_2_ and mercury acetate. It is also found in the form of organometallic cations such as methyl-, dimethyl-, and ethyl-mercury. In addition, mercury is also released into the air, water, and soil through anthropogenic activities [1].

It is well known that all forms of Hg are toxic. Acute exposure to Hg^0^ causes lung, kidney, and brain damage, while prolonged exposure to low levels causes neurological and psychiatric syndromes. Mercury is preferentially released into the environment as Hg^0^ vapor. In the atmosphere, it exhibits several transformations, predominantly its oxidation to mercury ions (mainly Hg^2+^), whereas in nature, mercury is mainly found as three different chemical species: elemental (Hg^0^), ionic (Hg^2+^) and organic (MeHg). The Hg released into the environment by both natural and anthropogenic sources reaches the marine ecosystem, where it is methylated by the action of microorganisms. The methylated form, MeHg, is rapidly taken up by living organisms in the aquatic environment and biomagnified through the food chain, reaching humans through the consumption of fish [2].

Human exposure to MeHg is global, as it is present in different concentrations in virtually all freshwater and marine organisms. Until the 1970s, organomercurials, especially MeHg and ethylmercury (EtHg), were widely used in agriculture as antifungal agents in cereal seeds [3]. Accordingly, the study and development of simple analysis techniques capable of speciating and quantifying trace amounts of mercury is a priority for many researchers [4,5,6].

Mercury determination involves two steps: a sample preparation and quantification. The sample preparation step is complicated due to the volatile character of mercury and its species [7]; however, the determination step is relatively simple and has often been carried out using the technique of vapor generation coupled to atomic absorption spectrometry (CVAAS), fluorescence spectrometry (CVAFS), inductively coupled plasma optical emission spectrometry (CVICP-OES) and coupled plasma mass spectrometry (CVICP-MS) [8,9,10].

Mercury has also been determined by electrothermal atomic absorption spectrometry (ETAAS) [11,12,13]. Due to the extraordinary volatility of the element, which has a significant vapor pressure even at room temperature, the direct use of ETAAS does not achieve a suitable sensitivity for this species because the concentration is usually very low. However, if a preconcentration procedure is used, the necessary sensitivity can be achieved taking advantage of the relative simplicity and convenience of this analytical technique which is present in most laboratories and is often underused [14].

In recent years, different microextraction techniques coupled with atomic absorption spectrometry have been used for the determination of Hg(II) [15,16,17]; however, very few works are found in the literature in which the speciation of mercury and its subsequent quantification are carried out [18]. Moreover, the analysis of MeHg in water samples is usually carried out using chromatographic techniques, although a derivatizing treatment of the sample is required [19,20]. Recently, the use of novel, hybrid sorption nanomaterials such as graphene oxide functionalized with ionic liquids (IL), and graphene-nickel functionalized with IL, have allowed the determination of Hg(II), MeHg and phenyl mercury (PhHg) without using chromatographic systems [21], although the separation of these materials from the analysis medium is difficult to complete. Efficient recoveries can be achieved by employing adsorbent materials exhibiting magnetic properties, such as graphitic carbon nitride [22].

In this work, an adsorbent with a large surface area and excellent characteristics, such as graphene oxide (GO) has been employed. The GO suspensions are so stable that its separation by a centrifugation step after the adsorption process is difficult; however, when GO forms a hybrid material with a magnetic compound such as Fe_3_O_4_, the hybrid product, a Fe_3_O_4_@GO composite, is easily separated from the solution by the application of a magnetic field. In the literature there are publications on the use of magnetic-reduced graphene oxide for Hg(II) retention and water purification, either without modification [23,24], or functionalized with EDTA [25]. Moreover, the development of these and other functionalized hybrid materials for the determination of Hg(II), both in water and food, has been recently reviewed [26]. While the analysis of Hg(II) has been extensively studied using these hybrid materials [27,28,29,30,31], however, the determination of MeHg has been scarcely implemented [32].

The main species of mercury in water are Hg(II) and mercury-organic species, in particular MeHg [33]. The latter has a strong bioaccumulation in living organisms. Moreover, the concentration of mercury in aquatic organisms is determined by its presence in the water, which in turn is related to the methylation and demethylation processes [34].

In this paper, a novel procedure is proposed based on a dispersive solid-phase microextraction using Fe_3_O_4_@GO as the adsorbent material and ETAAS as the technique for the determination of both Hg^2+^ and MeHg. The reliability of the approach has been verified by means of certified reference materials and then applied to the determination of mercury in water from different sources.

## 2. Results and Discussion

### 2.1. Effect of pH

**Figure 1:**
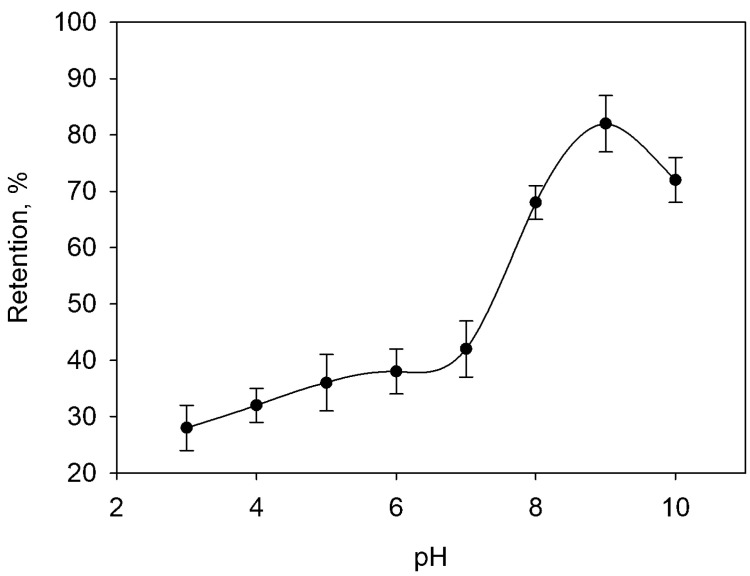
Effect of pH on Hg(II) retention. The error bars correspond to the standard deviation of three experiments.

The effect of pH on the adsorption of Hg(II) on Fe_3_O_4_@GO was studied by preparing suspensions with different acidity obtained by adding diluted HNO_3_, sodium hydroxide or buffer solutions. The results showed that the extent of the retention depended not only on the pH, but also on the main species present in the usual buffer solutions. The ability of Hg(II) to form complexes with common species such as chloride, hydroxyl, carbonate, sulfate and phosphate [35], ammonium [36] and dissolved organic matter [37] is known; consequently, the presence of these chemicals affects the determination of Hg(II) [38]. Due to the fact that in the development of the preliminary experiments the maximum retention was obtained at a pH slightly higher than 7, it was decided to use compounds that form buffer solutions lying in the range from 7 to 9 without interfering in the measurement of mercury, such as 2-amino-2-hydroxymethyl-propane-1,3-diol (TRIS), N-(2-hydroxyethyl) piperazine-N′-(2-ethanesulfonic acid) (HEPES), 2-[(2-hydroxy-1,1-bis(hydroxymethyl)ethyl)amino]ethanesulfonic acid (TES) and 2-(N-morpholino)ethanesulfonic acid (MES). The best outcomes were obtained with the use of TRIS as the main component of the buffer solution. Consequently, several suspensions were prepared, and the pH adjusted in the 3–10 range by adding sodium acetate-acetic acid or TRIS-HNO_3_ buffer solutions, as can be seen in Figure 1 where the results are summarized.

### 2.2. Retention of the Different Forms of Mercury

**Figure 2:**
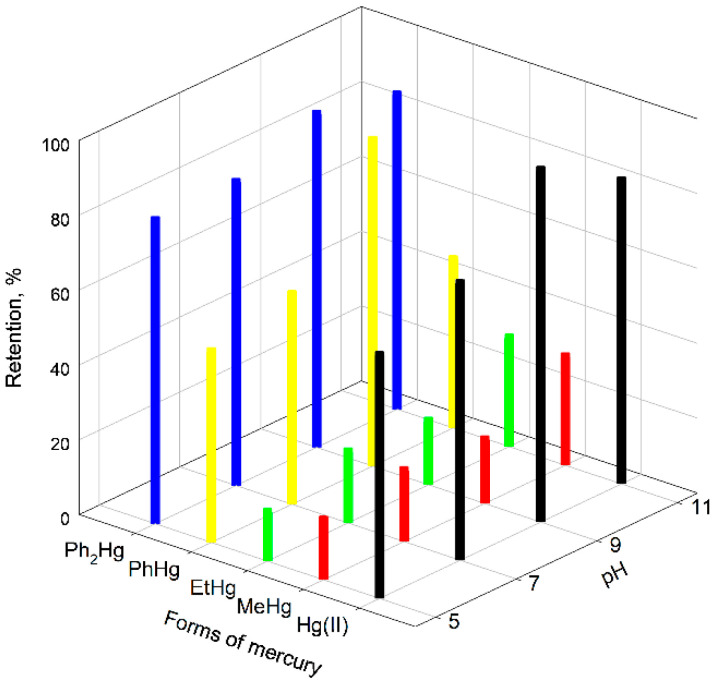
Retention of different forms of mercury in Fe_3_O_4_@GO at different pH values.

The retention of other mercury species that could eventually exist in the samples to be analyzed, namely, MeHg, EtHg, PhHg and diphenylmercury (Ph_2_Hg) was considered. A retention study of all of them on Fe_3_O_4_@GO was carried out at different pH values in the 5–11 range using the buffer solutions above commented. Figure 2 shows the percentage of retention of these chemicals in the different pH media. As can be seen, the greatest retention of mercury species occurred at a pH close to 9; however, EtHg and MeHg were hardly retained on Fe_3_O_4_@GO at any pH value.

**Figure 3:**
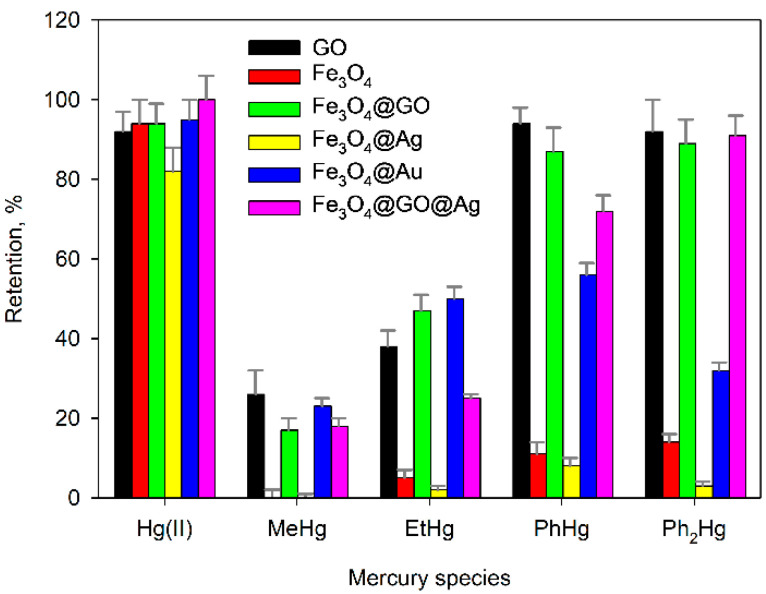
Retention of different forms of mercury in various adsorbent materials using TRIS as the buffer solution at pH = 9. Error bars correspond to the standard deviation of three experiments.

In order to achieve a reliable discrimination between Hg(II) and MeHg, a large number of experiments were carried out using other composites with magnetic properties. As Figure 3 shows, none of the sorbents assayed proved suitable for our purpose of speciation; therefore, a different strategy based in the use of auxiliary complexing agents was tried.

### 2.3. Effect of the Presence of Complexing Agents of Mercury Species

Chelating agents with thiol groups, such as L-Cysteine (L-Cys), sodium mercapto ethane sulfonate (MESNA) and 2,3-dimercaptopropanol (BAL) at different pH values, were assayed for the purpose. In the presence of L-Cys, the Hg(II) retention decreased and it was not even observed in the presence of MESNA and BAL.

However, when studying the effect of dithizone (1,5-diphenylthiocarbazone), a quantitative retention of all the mercury forms on the adsorbent material was observed. The amounts of dithizone and Fe_3_O_4_@GO to be used to achieve the total retention of Hg(II) and MeHg on the adsorbent were studied. The results showed that when 1 mL of a 0.05% *w*/*v* dithizone solution was employed, the retention of both forms of mercury was complete. A 100 µL amount of Fe_3_O_4_@GO suspension led to a maximum and constant retention of the mercury forms. In order to avoid that in the case of real samples, the presence of other species that can be adsorbed to saturate the active points of the adsorbent, the use of 300 µL of the Fe_3_O_4_@GO suspension is recommended. This volume involves 7 mg of adsorbent material per determination.

### 2.4. Study of Desorption Conditions

As mentioned above, all forms of mercury are retained in the presence of dithizone. To achieve the speciation of Hg(II) and MeHg, selective desorption media were studied. The study showed that if the magnetic particles were washed with N-acetyl-L-cysteine, MeHg was desorbed, while Hg(II) remained retained on the adsorbent material; however, this procedure was not considered reliable because when the concentration of N-acetyl-L-cysteine exceeded 0.05 M, the Hg(II) was also partially desorbed.

The strategy for the differentiation was then focused on the medium used for the adsorption process, since in the presence of dithizone both analytes were retained. Meanwhile, when a TRIS medium at pH = 9 was employed in the absence of dithizone, the Hg(II) was completely adsorbed together with a small fraction of the MeHg that could be easily released by the treatment with N-acetyl-L-cysteine.

In both adsorption procedures, the desorption of the species to be quantified by ETAAS was achieved in an acid medium. Hydrochloric acid is not recommended for use in atomic absorption spectrometry due to the formation of premature volatile species; therefore, HNO_3_ was employed for the purpose. Excellent results were obtained with the use of a mixture of 50 µL of 8% *v*/*v* HNO_3_ and 50 µL of 30% H_2_O_2_ and heating at 80 °C in an ultrasonic bath. After the treatment, the mixture was vortexed for 1 min to obtain a suspension, then, 10 µL aliquots were taken and injected into the atomizer, before applying the heating program shown in Table 1. For the Hg (II) determination, before adding the mixture of 8% *v*/*v* HNO_3_ and 50 µL of 30% H_2_O_2_, the adsorbent was washed with N-acetyl-L-cysteine to desorb the small amount of MeHg that might have been retained in the sorbent under those adsorption conditions.

### 2.5. Optimization of Analysis Conditions by ETAAS

At a first glance, taking into account the thermal and atomic characteristics of mercury, ETAAS does not appear to be a suitable quantification technique; however, a review of the literature revealed that by using the appropriate chemical modification, good sensitivity could be obtained. For example, it has been reported that the use of the mixture of Ag^+^ and MnO_4_^-^ led to excellent results in the determination of mercury in edible oils [18], waters [39], soils and sediments [40], sludges [41], infant foods [42] and food colorants [43]. Moreover, an interesting alternative is the use of metals such as palladium [13] or iridium [44] as permanent modifiers that are deposited on the walls of the graphite tube, either by electrolysis or by previously injecting several aliquots of the modifier [45].

It should be noted that in the procedure here studied, the suspension medium recommended caused the Pd(0) coating generated by electrolysis to be exhausted relatively quickly. A 20% loss of sensitivity in just 50 injections of 10 µL of the final suspensions was verified. An effective alternative was found in the prior injection of palladium and heating to a calcination temperature to achieve a deposition of the modifier on the surface of the L’Vov platform. It was found that the use of 20 µL of a 125 mg L^−1^ Pd(II) solution was sufficient to achieve mercury stabilization at 300 °C and its atomization at 1500 °C. Higher concentrations caused an excessive broadening of the signal that required too long integration times.

### 2.6. Calibration, Validation and Application to Real Samples

Applying the heating program recommended in Table 1, the analytical signal of Hg(II) and MeHg in the samples that were no submitted to the microextraction showed a linear behavior between 0.05 and 1 mg L^−1^ with a slope of 0.5814 s mg^−1^ L. The signal obtained after the application of the proposed procedure for the determination of the total mercury content showed a linear behavior between 0.1 and 10 µg L^−1^ with a calibration slope of 0.0284 s µg^−1^ L. The limit of detection (LOD), calculated as three times the standard error of the estimate, was 0.02 µg L^−1^, the limit of quantification (LOQ) was 0.06 µg L^−1^ and the average relative standard deviation for concentrations in the calibration interval was found to be 5.2%. The enrichment factor, calculated as the ratio of the slopes of a calibration line obtained in the procedure and the calibration line for the direct measurement of mercury was found to be 49. Extraction recovery (ER) is defined as the percentage of the total mercury amount which is extracted into the acceptor phase:

$$ER,\;\%=EF\times \frac{V_{e}}{V_{ac}}\times 100$$

where *V_e_* and *V_ac_* are the volumes of the acceptor phase and the aqueous sample, respectively. The ERs for the mercury ranged from 97–99%.

When the recommended procedure for the Hg(II) determination was applied, the calibration slope was 0.0281 s µg^−1^ L. The LOD and LOQ were 0.02 and 0.06 µg L^−1^, respectively, and the average relative standard deviation for concentrations in the calibration interval was found to be 4.7%, while the enrichment factor did not change.

The quantification was carried out by a direct calibration applying the proposed procedure to standard solutions of the analytes. No matrix effect was detected.

The reliability of the procedure was verified by analyzing the Hg(II) content in three reference materials: NCS DC 73347 (human hair), DORM-2 (fish muscle) and DORM-4 (fish protein) that were subjected to a microwave digestion process. In the NCS DC 73347, only the total mercury content was certified. Table 2 shows the results obtained. As can be seen, the concentrations found were consistent with the certified values.

In addition, another four standard reference samples, namely, NIST SRM 1640a (natural water with a low salt content), SPS-SW2 (surface water reference material), ERM CAO11b (reference material), and NASS-6 (sea water) were also submitted to the recommended procedure. It is of note that these water samples did not have a certified mercury content. In this case, the standard addition method was applied. The results are summarized in Table 3.

The procedure proposed for the determination of the total Hg and Hg(II) was also applied to the determination of these species in the water samples from different sources. As shown in Table 4, a recovery test was applied because the signal obtained in the samples was below the detection limit.

Table 5 shows a comparison of the proposed procedure with others that, using different microextraction techniques, have in common the determination of mercury by ETAAS. As can be seen, the calibration interval and LOD were comparable to, or even better than many of the procedures listed in the table. The sample consumption in the determination was very low, the adsorbent material was easy to prepare and the magnetic separation facilitates the experimental procedure and the determination of the two main mercury species in water.

## 3. Materials and Methods

### 3.1. Materials and Instrumentation

All experiments were carried out using high purity water (resistivity 18 MΩ cm) obtained with a Millipore system (Millipore, Bedford, MA, USA). The Hg(II) standard solution (1000 mg L^−1^ in 1% HNO_3_, 250 mL) was purchased from Fluka (Buchs, Switzerland). The standard solution of MeHg 1000 mg L^−1^ was prepared by dissolving the appropriate amount of its chloride (Merck, Darmstadt, Germany) in the smallest possible volume of methanol and diluting to volume with water. Standard solutions of 100 mg mL^−1^ of EtHg, PhHg and Ph_2_Hg were prepared in the minimum amount of acetone or hexane from the products also supplied by Sigma. Working standard solutions were obtained by an appropriate dilution and were prepared daily by dilution with ultrapure water. Caution: all forms of mercury are toxic and suitable precautions must be taken when handling them [54,55,56]. Mercury poisoning can result from inhalation, ingestion, and injection or absorption through the skin. All forms of mercury penetrate the placental barrier and should be considered teratogenic and reproductive effectors. The effects may not be noticeable for months or years. Inorganic mercury salts are toxic (LD50 from 6–200 mg kg^−1^). Dimethyl mercury is even more toxic (LD50: 50 µg kg^−1^) and extreme caution is required when working with this material.

The solutions of dithizone, L-cysteine, N-acetyl-L-cysteine, 2-Amino-2-hydroxymethyl-propane-1,3-diol (TRIS), tetramethyl ammonium hydroxide (TMAH), and other reagents were prepared from reagents supplied by Sigma-Aldrich. The dithizone solution was prepared by dissolving, with the help of ultrasound, 0.05 g of the product in 100 mL of a 4:1 (*v*/*v*) mixture of acetone and an aqueous solution adjusted to a pH = 9 with ammonium hydroxide.

The Fe(II) and Fe(III) salts used were FeCl_2_·4H_2_O and FeCl_3_·6H_2_O, respectively, which were obtained from Sigma. The graphene oxide was provided by Timesnano (Chengdu Organic Chemicals Co. Ltd., Chinese Academy of Sciences, Chengdu, China). The manufacturer’s specifications indicate a purity > 98%, ash < 1.5%, 1–2 layers and a 1–5 µm of diameter. The atomic percentage of C and O are 64.71 and 35.29, respectively.

The materials used as the water reference samples were SRM 1640a (trace elements in natural water) from the National Institute of Standards and Technology (Gaithersburg, MD, USA), SRMs SPS-SW2 (surface water) and ERM CAO11b (natural water) from LGC Limited (Teddington, UK), and the NASS-6 material (seawater) from the National Research Council of Canada, Ottawa, Canada (NRC-CNRC).

To verify the reliability of procedures, the results obtained in the determination of the total mercury, Hg(II) and MeHg were compared with certified reference materials: DORM-4 (fish protein), DORM-2 (fish muscle) and DOLT-2 (fish liver), supplied by the NRC-CNRC, as well as the material DC 73347 (human hair) that was supplied by the China National Analysis Center for Iron and Steel, Beijing, P. R. China.

An Analytik Jena ContrAA 700 high-resolution atomic absorption spectrometer, equipped with a 300 W xenon short-arc lamp as a continuous radiation source (Analytik Jena, Germany) was used to carry out all the measurements. A transversely heated graphite atomizer with pyrolytically coated graphite tubes and a L’Vov platform was used for the analyte atomization. The data were evaluated using the summed integrated absorbance for 3, 5 or 7 pixels using the software (ASPECT CS v5.1) provided by Analytik Jena.

For the ultrasonic treatment, a 50 W thermostatted ultrasound bath (ATU, Valencia, Spain) was employed. In the desorption species process, a vortex, Reax model from Heidolph (Schwabach, Germany) was used. The digestion of the reference materials was carried out with a microwave oven (Multiwave 3000 Anton-Paar, Perkin Elmer, Shelton, CT, USA) equipped with pressure and temperature control.

### 3.2. Preparation of Fe_3_O_4_@GO

The Fe_3_O_4_@GO was prepared by the precipitation of Fe(II) and Fe(III) salts in the presence of graphene oxide and ammonium hydroxide. Firstly, a suspension of 0.1 g of graphene oxide in 50 mL of water was prepared and sonicated for one hour. Then, under a nitrogen atmosphere, 10 mL of FeCl_3_·6H_2_O (0.1475 g mL^−1^) and FeCl_2_·4H_2_O (0.046 g mL^−1^) solutions were added dropwise. The mixture was heated at 80 °C while being stirred for 10 min. Concentrated ammonium hydroxide was then added slowly until reaching a pH = 11. In this way, a black precipitate of Fe_3_O_4_ particles was obtained. The magnetic particles were separated with the help of an external magnet. The supernatant was decanted and discarded. The magnetic particles were washed 3 times with 25 mL aliquots of ethanol in order to remove the excess salts and ions. Finally, they were suspended in 30 mL of water. The estimated concentration of Fe_3_O_4_@GO obtained was 24 mg mL^−1^.

### 3.3. Sample Treatment

For the determination of the total mercury in the reference materials, 0.5 g of this material was placed into a digestion vial. Then, 4.0 mL of HNO_3_ acid and 4 mL of 30% *v*/*v* H_2_O_2_ were added. The vial was placed into the microwave oven and a power of 1400 W was applied for 20 min. At the end of the digestion program, the mixture was cooled down before being removed from the microwave unit (about 15 min). Finally, the pH was adjusted to 5.0 by the addition of a concentrated NaOH solution and the liquid was diluted to 25 mL with pure water in a volumetric flask.

For the determination of Hg(II) and MeHg, a procedure based on previous studies was carried out [57,58]. To achieve the solubilization of these mercury species, 0.5 g amounts of the certified reference materials were accurately weighed into a conical tube. Then, 2 mL of a 25% (*m*/*v*) TMAH solution were added to the sample and the mixture was first kept at room temperature for three hours and then heated in a water bath at 80 °C for 30 min. Finally, the mixture was cooled and diluted to 25 mL with pure water.

The water samples were filtered immediately after collection using a cellulose nitrate filter membrane (with a 0.45 µm pore size). Subsequently, they were stored in low-density polypropylene bottles at 4 °C until analysis within 3 days.

### 3.4. Procedure for the Determination of Total Mercury

A 10 mL sample was placed in a 15 mL tube, and 400 µL of a 0.1 M acetate/acetic buffer (pH = 5) and 1 mL of a 0.05% *w*/*v* dithizone solution were added. The solution was manually homogenized and 300 µL of the Fe_3_O_4_@GO suspension was added. The mixture was stirred for 10 min. Afterwards, the magnetic particles were separated by action of the magnet and the supernatant liquid was discarded. The solid residue was suspended in 50 µL of HNO_3_ at 8% *v*/*v* and 50 µL of H_2_O_2_ at 30% *v*/*v* using ultrasound into a thermostatic bath at 80 °C for 10 min.

An amount of 10 µL of this suspension was injected into the atomizer where 20 µL of a Pd(II) solution at 125 mg L^−1^ was previously injected. The heating program shown in Table 1 was applied, thus obtaining an analytical signal corresponding to the total mercury content in the sample.

**Figure 4:**
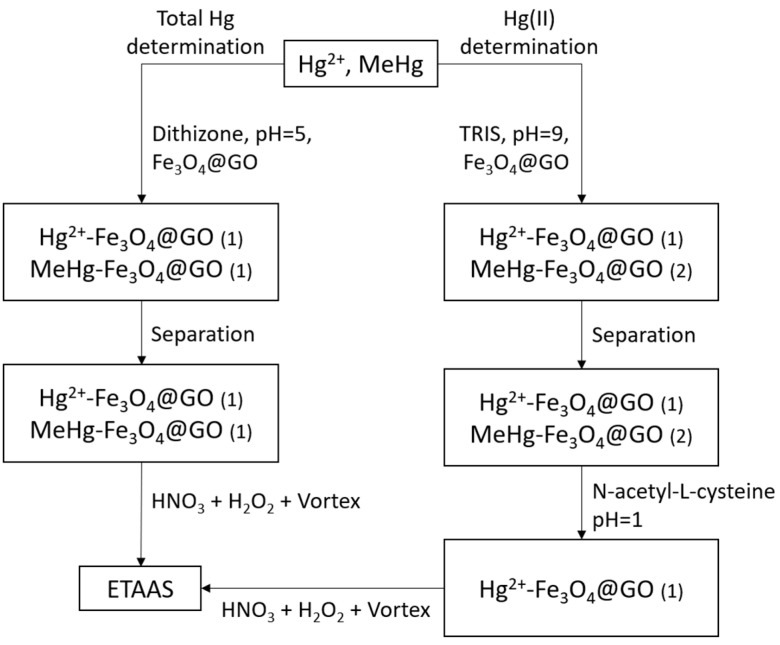
Scheme of the process from extraction to analysis. Complete and incomplete retentions of the analytes are represented by (1) and (2), respectively.

Figure 4 shows a scheme of the extraction process. Complete and incomplete retentions of the analytes are represented by (1) and (2), respectively.

### 3.5. Procedure for the Determination of Hg(II)

A 300 µL volume of a 0.1 mol L^−1^ buffer solution (TRIS/HNO_3_ with a pH = 9) was added to 10 mL of water. The solution was mixed and 300 µL of the Fe_3_O_4_@GO suspension was added then the mixture was stirred for 10 min. Next, the magnetic nanoparticles were separated by a magnet and the supernatant liquid was discarded. Afterwards, the particles were washed with 1 mL of 0.01 M N-acetyl-L-cysteine. Finally, the magnetic particles were suspended in 50 µL of HNO_3_ at 8% *v*/*v* and 50 µL of H_2_O_2_ at 30% *v*/*v* using ultrasound in a thermostatic bath at 80 °C for 10 min.

A 10 µL volume of this suspension was injected with 20 µL of the Pd(II) solution at 125 mg L^−1^ and the heating program shown in Table 1 was applied. The measured signal corresponded to the content of Hg(II) in the sample.

### 3.6. Procedure for the Determination of MeHg

Since the level of organomercurials different to MeHg was considered negligible for the samples considered, the content of MeHg was estimated as the difference between the total content of total mercury and the content of Hg(II).

## 4. Conclusions

A novel procedure based on a dispersive solid-phase microextraction with Fe_3_O_4_@GO as the adsorbent material for the determination of different forms of mercury is proposed. For this approach, it is not necessary to resort to sophisticated and relatively expensive analysis techniques, instead it employs a robust and economical methodology accessible to many laboratories such as ETAAS. The coupling of an adequate microextraction technique allowed for reaching the necessary detection limits for mercury and the validation of the method of analysis of this species. The proposed procedure presents an enrichment factor of 49 and the determination presents a linear interval between 0.1 and 10 µg L^−1^ of mercury. Furthermore, the method was successfully applied to the determination of mercury in waters of different origins and reference materials.

## Figures and Tables

**Table 1:** Instrumental parameters and heating program for mercury determination.

Parameter			
Wavelength, nm		253.6519	
Slit, nm		0.7	
Atomizer		Transversal with L’Vov platform	
Background correction		Zeeman effect	
Injected sample volume, µL		10	
Chemical modifier		20 µL of 125 mg L^−1^ Pd(II) solution	
Sample volume, mL		10	
Heating program			
Step	Temperature, °C	Ramp, °C s^−1^	Hold, s
1: Dry	110	10	30
2: Pyrolysis	300	50	20
4: Atomization ^a,b^	1500	2200	5
5: Clean	1500	0	4
Sequence: Inject chemical modifier and run steps 1 to 2. Stop heating and inject sample. Then run the heating program.			

^a^ Internal argon flow stopped 5 s before. ^b^ Reading step.

**Table 2:** Total mercury, Hg(II) and MeHg contents in hair and fish reference materials (SRM).

	Total Hg, µg g^−1^		MeHg ^a^, µg g^−1^		Hg(II) ^a^, µg g^−1^	
SRM	Certified	Found	Certified	Found	Certified	Found
NCS DC73347	0.360 ± 0.050	0.37 ± 0.07	--	0.37 ± 0.07	--	<LOD
DORM-4	0.410 ± 0.055	0.408 ± 0.06	0.355 ± 0.028	0.348 ± 0.04	--	0.06 ± 0.03
DORM-2	4.64 ± 0.26	4.67 ± 0.12	4.47 ± 0.32	4.42 ± 0.06	--	0.26 ± 0.06
DOLT-2	2.14 ± 0.18	2.18 ± 0.09	--	1.49 ± 0.09	--	0.693 ± 0.08

^a^ mean value in Hg ± standard deviation of three digestions.

**Table 3:** Total mercury, Hg(II) and MeHg in water reference materials (SRM).

	Hg Total, µg L^−1^			MeHg ^a^, µg L^−1^			Hg(II) ^a^, µg L^−1^		
SRM	Added	Found	Rec. %	Added	Found	Rec. %	Added	Found	Rec. %
NIST SRM 1640a	0	<LOD	--	0	<LOD	--	0	<LOD	--
	7	6.9 ± 0.1	98 ± 3	2	1.9 ± 0.1	96 ± 2	5	5.0 ± 0.1	99 ± 3
	7	7.3 ± 0.1	105 ± 3	5	5.1 ± 0.2	103 ± 3	2	2.2 ± 0.1	110 ± 5
SPS-SW2	0	<LOD	--	0	<LOD	--	0	<LOD	--
	7	6.9 ± 0.1	98 ± 3	2	1.9 ± 0.1	96 ± 3	5	4.9 ± 0.1	99 ± 3
	7	6.8 ± 0.2	97 ± 2	5	4.9 ± 0.1	98 ± 3	2	1.9 ± 0.1	95 ± 4
ERM CAO11b	0	<LOD	--	0	<LOD	--	0	<LOD	--
	7	7.1 ± 0.1	102 ± 3	2	2.1 ± 0.1	104 ± 4	5	5.0 ± 0.2	101 ± 3
	7	7.2 ± 0.1	103 ± 2	5	5.1 ± 0.2	103 ± 3	2	2.0 ± 0.2	101 ± 4
NASS-6	0	<LOD	--	0	<LOD	--	0	<LOD	--
	7	6.9 ± 0.2	99 ± 3	2	1.9 ± 0.1	95 ± 3	5	5.0 ± 0.2	101 ± 3
	7	6.9 ± 0.1	99 ± 2	5	5.0 ± 0.1	100 ± 3	2	1.9 ± 0.1	96 ± 3

^a^ mean value in Hg ± standard deviation of three determinations.

**Table 4:** Total mercury, Hg(II) and MeHg in real water samples (RWS).

	Hg Total. µg L^−1^			MeHg ^a^. µg L^−1^			Hg(II) ^a^. µg L^−1^		
RWS	Added	Found	Rec. %	Added	Found	Rec. %	Added	Found	Rec. %
M1	0	<LOD	--	0	<LOD	--	0	<LOD	--
	4	3.92 ± 0.09	97.9 ± 5.3	2	1.97 ± 0.04	98.5 ± 2.3	2	1.95 ± 0.03	97.3 ± 1.8
M2	0	<LOD	--	0	<LOD	--	0	<LOD	--
	4	3.80 ± 0.03	95.1 ± 2.3	2	1.91 ± 0.07	95.5 ± 4.1	2	1.89 ± 0.07	94.5 ± 4.1
M3	0	<LOD	--	0	<LOD	--	0	<LOD	--
	4	4.01 ± 0.06	100.3 ± 4.2	2	2.00 ± 0.06	100.0 ± 3.2	2	2.01 ± 0.02	100.6 ± 1.7
M4	0	<LOD	--	0	<LOD	--	0	<LOD	--
	4	4.02 ± 0.05	100.6 ± 4.0	2	2.02 ± 0.05	100.9 ± 2.6	2	2.01 ± 0.05	100.3 ± 2.6
M5	0	<LOD	--	0	<LOD	--	0	<LOD	--
	4	3.89 ± 0.01	97.2 ± 2.1	2	1.89 ± 0.06	94.5 ± 3.1	2	2.00 ± 0.04	99.9 ± 2.4
M6	0	<LOD	--	0	<LOD	--	0	<LOD	--
	4	3.67 ± 0.07	91.8 ± 3.9	2	1.88 ± 0.07	93.9 ± 4.1	2	1.79 ± 0.01	89.7 ± 1.1
M7	0	<LOD	--	0	<LOD	--	0	<LOD	--
	4	3.99 ± 0.06	99.7 ± 4.2	2	2.07 ± 0.03	103.5 ± 1.9	2	1.92 ± 0.03	95.8 ± 1.7
M8	0	<LOD	--	0	<LOD	--	0	<LOD	--
	4	4.11 ± 0.02	102.6 ± 3.7	2	2.08 ± 0.07	103.8 ± 4.0	2	2.03 ± 0.09	101.4 ± 5.1

M1: drinking water from the supply network; influent water (M2) and effluent (M3) from a wastewater treatment plant; M4: water from a natural source; M5: snow water; M6: water from the Segura river; M7: water from the La Cierva reservoir (Mula, Murcia, Spain); M8: water from the Mar Menor. ^a^ mean value in Hg ± standard deviation of three digestions.

**Table 5:** Some recent procedures for ETAAS determination and/or speciation of mercury using microextraction techniques.

Specie	Microextraction Technique	Remarks	CM	T, °C	L, µg L^−1^	LOD, µg L^−1^	EF	Ref.
Hg(II), MeHg, PhHg	SPE with MOF	Thermal release.	None	135–275/800	0.001–0.5 ^a^	0.06	--	[11]
Hg(II), MeHg	Double CPE	1: dithizone; 2: thiourea	Pd(II)	200/1800	0.4–15	0.23	17.8	[46]
MeHg; Hg(II)	Double HF-LPME	1: MeHg (S_2_O_3_^=^); 2: Hg(II) (DDTC).	Pd(II)	200;120/ 1800; 1300	0.5–8; 0.2–12	0.143; 0.063	103; 95	[47]
MeHg; Hg(II)	SFODME in two steps	1: MeHg (1-undecanol); 2: MeHg (4-NODP).	Pd(II)	250/1300	0.8–8	0.24; 0.25	32.2; 25.7	[48]
Hg(II)	LPME with eutectic solvent	DDTP.	Ir(IV)	210/1100	0.36–60	0.1	98	[49]
Hg(II)	SPME with GO + (C_4_C_12_Im)Br	Methodology in flow and elution with HNO_3_.	Pd(II)	250/1300	0.02–8	0.014	100	[50]
Hg(II)	SPME with Fe_3_O_4_@SiO_2_@DPTH	Methodology in flow and elution in thiourea.	Ir(0)	20/1200	0.1–10	0.0078	5.4	[44]
Hg(II); MeHg, Me_2_Hg, EtHg, PhHg,Ph_2_Hg	DSPME with Fe_3_O_4_@Ag@MESNA or Fe_3_O_4_@Ag@CYS	1: Fe_3_O_4_@Ag@MESNA for Hg(II); 2: Fe_3_O_4_@Ag@CYS for others.	Ag(I) + KMnO_4_	300/1300	0.03–3.5	0.01	200	[18]
Hg(II)	AgNPs and DSPME	The amalgamated Hg is dissolved in HNO_3_.	Pd(II) + Mg(II)	300/1300	0.1–20	0.005	15	[51]
Hg(II)	HS-SDME	Hg(0) with SnCl_2_ and drop of Pd(0).	Pd(II)	200/1300	1.5–40	0.48	75	[52]
Hg(II)	DSPME with PdNPs functionalized with dodecanethiolate	The nanoparticles are in a mixture of toluene and chloroform.	Pd(II)	250/1300	0.1–10	0.0075	95	[17]
Hg(II), MeHg	HS-SDME with thiourea or APDC	Generation of Hg(0) and MeHg hydride with NaBH_4_.	--	250/1100	17–355	5	35	[53]
Hg(II)	HF-LPME three-phase	1: complexation of Hg(II) with PAN; 2: toluene extraction; 3: iodide back extraction.	Pd(0)	300/1100	0.2–3	0.06	270	[13]
Hg(II), MeHg	DSPME with Fe_3_O_4_@GO	1: Dithizone for total Hg; 2: NAC for Hg(II).	Pd(II)	300/1500	0.1–10	0.02	49	TW

CM: chemical modifier; T: ash and atomization temperatures; EF: enrichment factor; SPE: solid-phase extraction; MOF: metal organic framework; CPE: cloud point extraction; HF-LPME: hollow fiber liquid phase microextraction; DDTC: diethyldithiocarbamate; 4-NODP: 4-nitro-o-fenilen diamine; DDTP: diethyldithiophosphate; (C_4_C_12_im)Br: 1-butil 3-dodecilimidazolium bromide; Fe_3_O_4_@SiO_2_@DPTH: ferrite-silica-1,5-bis(di-2pyridyl)methylene thiocarbohydrazide nanocomposite; MESNA: sodium 2-mercapto ethane sulfonate; CYS: cysteine; HS-SDME: head space single drop microextraction; DSPME: dispersive solid-phase microextraction; NAC: N-acetyl-L-cysteine; TW: this work. ^a^ mean value in Hg ± standard deviation of three digestions.

## Data Availability

Data are available from the corresponding authors upon reasonable request.
